# Supplementary material for: MetaRibo-Seq measures translation in microbiomes
Source: Nat Commun. 2020 Jun 29;11:3268. doi: 10.1038/s41467-020-17081-z (PMC7324362; doi:10.1038/s41467-020-17081-z)
Supplement: Supplementary file 10 — Supplementary Data 7 [file 41467_2020_17081_MOESM10_ESM.zip › File2/Confidence_High_Taxonomy/13732_out.krona.html]

Javascript must be enabled to view this page.

members
magnitude
magnitudeUnassigned
count
unassigned
taxon
rank

13732\_out

4

superkingdom
4
2

4
1239
phylum

186801
4
class

order
4
186802


SRS047433\_contig\_number\_23715SRS058770\_contig\_number\_20863SRS077502\_contig\_number\_12578SRS144537\_contig\_number\_13409
species
1897049
4
